# RGD-based self-assembling nanodrugs for improved tumor therapy

**DOI:** 10.3389/fphar.2024.1477409

**Published:** 2024-10-01

**Authors:** Bin Wang, Dongmei Tang, Jianqiao Cui, Hongfei Jiang, Jing Yu, Zhu Guo

**Affiliations:** ^1^ Department of Sports Medicine, Affiliated Hospital of Qingdao University, Qingdao University, Qingdao, China; ^2^ The Affiliated Hospital of Qingdao University, Qingdao University, Qingdao, China; ^3^ Qingdao Hospital, University of Health and Rehabilitation Sciences, Qingdao, Municipal Hospital, Qingdao, China; ^4^ Department of Spinal Surgery, Affiliated Hospital of Qingdao University, Qingdao, China

**Keywords:** RGD peptides, self-assembling nanodrugs, tumor targeting, cancer therapy, integrin receptors

## Abstract

RGD-based self-assembling nanodrugs are a promising advancement in targeted cancer therapy, combining the specificity of RGD peptides with the benefits of nanotechnology. These nanodrugs enhance tumor targeting and cellular uptake while reducing off-target effects. RGD peptides facilitate the self-assembly of stable nanostructures, ensuring efficient drug delivery. Despite their potential, challenges such as immunogenicity, stability, tumor heterogeneity, and manufacturing scalability need to be addressed. Future research should focus on improving biocompatibility, advanced targeting strategies, personalized medicine approaches, and innovative manufacturing techniques. Overcoming these challenges will pave the way for the successful clinical translation of RGD-based nanodrugs, offering more effective and safer cancer treatments.

## 1 Introduction

Cancer remains a major global health challenge, responsible for millions of deaths each year. Traditional chemotherapy, while effective, often suffers from significant limitations including non-specific distribution, severe side effects, and drug resistance (Fang et al., 2023; Zhong et al., 2021; Yan et al., 2024; Boumahdi and de Sauvage, 2020). These issues have prompted the development of advanced drug delivery systems designed to enhance the efficacy and safety of cancer treatments (Agiba et al., 2024; Zhao et al., 2023; Zhang et al., 2023a; Liu N. et al., 2023; Zhang et al., 2023b; Tu et al., 2024). One such promising approach involves the use of nanotechnology to create self-assembled nanodrugs (Tian and Lu, 2022; Lu et al., 2024; Qiao et al., 2022).

Nanodrugs leverage the unique properties of nanoparticles to improve drug delivery. They can encapsulate chemotherapeutic agents, protecting them from degradation, enhancing their solubility, and allowing for controlled release (Chen et al., 2022; van der Meel et al., 2019; Haist et al., 2022). Among various strategies, self-assembled nanodrugs have gained particular attention due to their ability to form well-defined structures through spontaneous organization of their components (Huang et al., 2021; Liu Y. et al., 2023; Li et al., 2024). This process, driven by non-covalent interactions such as hydrophobic effects, electrostatic interactions, and hydrogen bonding, enables the precise engineering of nanoparticle size, shape, and functionality.

A critical aspect of effective cancer therapy is the ability to target tumor cells specifically, thereby minimizing harm to healthy tissues (Leng et al., 2024; Liu et al., 2024). This is where RGD (arginine-glycine-aspartic acid) peptides come into play. RGD peptides are known to bind with high affinity to several integrin receptors, including αvβ1, αvβ3, αvβ5, αvβ6, αvβ8, α5β1, αIIbβ3, and α8β1, all of which play crucial roles in tumor biology and beyond. Although αvβ3 and αvβ5 integrins have been extensively studied in tumor targeting, emerging research highlights the importance of other RGD-binding integrins such as α5β1 and αvβ6, which may offer more specific and effective targeting strategies in cancer therapy (Kesharwani et al., 2024; Sanati et al., 2023; Nader et al., 2020; Rana et al., 2024). It is important to note that αvβ3 integrin is not exclusively expressed by tumor cells. It is also found in various physiological processes, particularly in the vasculature, making tumor-specific targeting using αvβ3-directed approaches challenging. This poses a limitation in achieving selective tumor accumulation and may lead to off-target effects (Hosseinikhah et al., 2024; Sheikh et al., 2021).

Integrin receptors play a crucial role in tumor angiogenesis, metastasis, and survival, making them attractive targets for cancer therapy (Liu F. et al., 2023; Marelli et al., 2013; Moreno-Layseca et al., 2019). The RGD motif mimics the natural ligands of these receptors, allowing for selective binding and internalization by tumor cells. This targeted approach not only improves the concentration of the drug at the tumor site but also reduces systemic toxicity and adverse side effects (Van Hove et al., 2021; Slack et al., 2022).

Recent advances in nanotechnology and peptide engineering have enabled the development of sophisticated RGD-based self-assembled nanodrugs (Kesharwani et al., 2024). These nanodrugs combine the targeting capabilities of RGD peptides with the versatile drug delivery properties of nanoparticles. They can be engineered to encapsulate a wide range of therapeutic agents, including small-molecule drugs, proteins, and nucleic acids, and deliver them directly to tumor cells.

In summary, RGD-based self-assembled nanodrugs represent a cutting-edge approach in cancer therapy. By harnessing the specificity of RGD peptides and the advantages of self-assembled nanostructures, these nanodrugs offer the potential for more effective and safer cancer treatments. The following sections will explore the mechanisms of RGD-based targeting, the process of self-assembly, the therapeutic benefits, and the recent advancements and future prospects in this exciting field.

## 2 Distinct advantages of RGD-based self-assembled nanodrugs

RGD-based self-assembled nanodrugs combine the advantages of targeted delivery with the benefits of nanotechnology, resulting in a highly effective therapeutic approach for cancer treatment. The RGD peptides play crucial roles in both tumor targeting and the self-assembly process, leading to unique advantages. Here, we detail the structure and distinct benefits of RGD-based self-assembled nanodrugs.

### 2.1 Types and variations of RGD

RGD peptides can exist in several forms and configurations, including linear and cyclic versions (Li et al., 2022). The primary types of RGD peptides include linear RGD peptides and cyclic RGD peptides. Linear RGD peptides are straightforward to synthesize and modify. Alternatively, the cyclic RGD are cyclized through the formation of a disulfide bond or through head-to-tail cyclization, enhancing their stability and binding affinity by reducing the flexibility of the molecule. RGD peptides exist in various forms, including linear, cyclic, and multivalent configurations. Linear RGD peptides, while easy to synthesize, tend to be less stable in physiological environments. Cyclic RGD peptides, such as c(RGDfK), c(RGDyK), c(RGDyC), c(RGDfC), and iRGD, where “f” denotes D-phenylalanine, “y” denotes tyrosine, and “C” denotes cysteine, providing additional stabilization through disulfide bonds, offer improved stability and binding affinity due to their conformational rigidity. In addition to these, multivalent RGD peptides, which present multiple RGD motifs in a single structure, are being explored to enhance binding specificity and avidity for integrin-rich tumor cells, further expanding the toolbox for integrin-targeted therapies (Park et al., 2012; Sani et al., 2021). Notably, studies have shown that cyclic RGD peptides (cRGD) exhibit superior binding efficacy compared to linear RGD peptides due to their enhanced stability and conformational rigidity. Fu et al. (2021) demonstrated that liposomes modified with c (RGDfK) exhibited significantly higher cellular uptake and tumor inhibition in lung cancer models than those with linear RGD. Moreover, Bogdanowich-Knipp et al. (1999) found that cyclic peptides, such as cyclo-(1,6)-Ac-Cys-Arg-Gly-Asp-Phe-Pen-NH2, displayed greater stability, particularly at neutral pH, contributing to their prolonged biological activity and enhanced integrin binding.

### 2.2 Tumor targeting ability of RGD peptides

#### 2.2.1 High affinity and specificity

RGD peptides are known for their strong binding affinity to integrin receptors, particularly αvβ3 and αvβ5, which are overexpressed on the surface of various tumor cells and their vasculature. This high specificity ensures that RGD-based nanodrugs selectively accumulate in tumor tissues, enhancing the local concentration of the therapeutic agent and reducing systemic exposure. Recent studies suggest that integrins such as α5β1 and αvβ6, which are involved in critical tumor processes such as invasion, metastasis, and survival, may offer better specificity for tumor targeting. α5β1, in particular, plays a key role in fibronectin-mediated cell adhesion, making it an attractive target in aggressive tumor types. Similarly, αvβ6 integrin, which is upregulated in many epithelial cancers, has been identified as a potential marker for tumor progression and metastasis, providing a more selective targeting option in certain cancer types (Zhou et al., 2023; Niu and Li, 2017).

#### 2.2.2 Enhanced cellular uptake

The interaction between RGD peptides and integrin receptors facilitates receptor-mediated endocytosis, significantly increasing the internalization of nanodrugs by tumor cells. This enhanced uptake ensures that a larger proportion of the drug reaches its intracellular targets, thereby improving its therapeutic efficacy. In addition to the design aspects of RGD-modified NPs, comparative studies between RGD-modified and non-RGD-modified nanoparticles have demonstrated significant differences in cellular uptake and tumor targeting. RGD peptides, particularly in their cyclic forms, significantly enhance the interaction with integrin receptors (such as αvβ3 and αvβ5) that are overexpressed in tumor cells. For example, studies have shown that RGD-modified NPs exhibit increased cellular internalization through receptor-mediated endocytosis, resulting in higher drug accumulation in tumor tissues. In contrast, non-RGD-modified NPs primarily rely on passive targeting, which limits their cellular uptake and reduces their overall therapeutic efficacy (Chen et al., 2012).

#### 2.2.3 Reduced off-target effects

By specifically targeting integrin receptors that are primarily expressed on tumor cells, RGD-based nanodrugs minimize interactions with normal tissues. This selective targeting reduces the adverse side effects commonly associated with conventional chemotherapy, leading to a better safety profile. While RGD-modified NPs exhibit superior targeting and cellular uptake, their biocompatibility and toxicity profiles are equally important for clinical translation. The biocompatibility of RGD-modified NPs has been shown to be generally favorable, with minimal immune response in short-term studies. However, long-term biocompatibility and potential toxicity, especially due to repeated administration, require further investigation. Some studies suggest that the use of cyclic RGD peptides reduces immunogenicity due to their stability, but systemic toxicity remains a concern if off-target interactions occur (Xu et al., 2023; Wang et al., 2022).

### 2.3 Self-assembly promotion of RGD peptides

#### 2.3.1 Facilitated nanostructure formation

RGD peptides can influence the self-assembly process by promoting the formation of well-defined nanostructures. Their amphiphilic nature, with both hydrophilic and hydrophobic regions, assists in the spontaneous organization of drug molecules into stable nanoparticles through non-covalent interactions such as hydrophobic effects and hydrogen bonding.

#### 2.3.2 Enhanced stability and solubility

The incorporation of RGD peptides into nanodrug formulations can improve the solubility and stability of hydrophobic chemotherapeutic agents. Self-assembled nanodrugs protect these agents from premature degradation and enhance their solubility in aqueous environments, facilitating their delivery to the tumor site.

#### 2.3.3 Controlled drug release

Self-assembled nanodrugs can be engineered to provide controlled and sustained release of the encapsulated therapeutic agents. The presence of RGD peptides in the nanostructure can influence the release kinetics, ensuring a steady and prolonged delivery of the drug, which can enhance its therapeutic efficacy while minimizing peak-related toxicity.

#### 2.3.4 Multifunctional nanocarriers

RGD-based self-assembled nanodrugs offer the potential for multifunctionality. They can be designed to carry multiple therapeutic agents, imaging agents, or targeting moieties within a single nanocarrier. This multifunctionality enables simultaneous cancer treatment and diagnosis (theranostics), providing a comprehensive approach to cancer management.

In summary, the distinct advantages of RGD-based self-assembled nanodrugs lie in their ability to combine targeted delivery with the efficient encapsulation and controlled release of therapeutic agents. The RGD peptides not only enhance tumor specificity and cellular uptake but also promote the self-assembly of stable and functional nanostructures. These unique benefits make RGD-based self-assembled nanodrugs a promising approach for improving the outcomes of cancer therapy.

## 3 Recent advances and applications

Recent studies have demonstrated the efficacy of RGD-based self-assembled nanodrugs in various cancer models, showing significant improvements in targeting, penetration, and therapeutic outcomes. These nanodrugs leverage the high affinity of RGD peptides for integrin receptors, facilitating precise delivery to tumor cells and reducing off-target effects. Advances in nanotechnology have enabled the development of multifunctional nanodrugs that combine chemotherapy, phototherapy, and imaging capabilities. *In vitro* and *in vivo* experiments have confirmed their potential in enhancing drug accumulation at tumor sites, improving cellular uptake, and achieving sustained drug release, ultimately leading to superior antitumor activity and reduced systemic toxicity.

### 3.1 Linear RGD-based self-assembling nanodrugs

**FIGURE 1:**
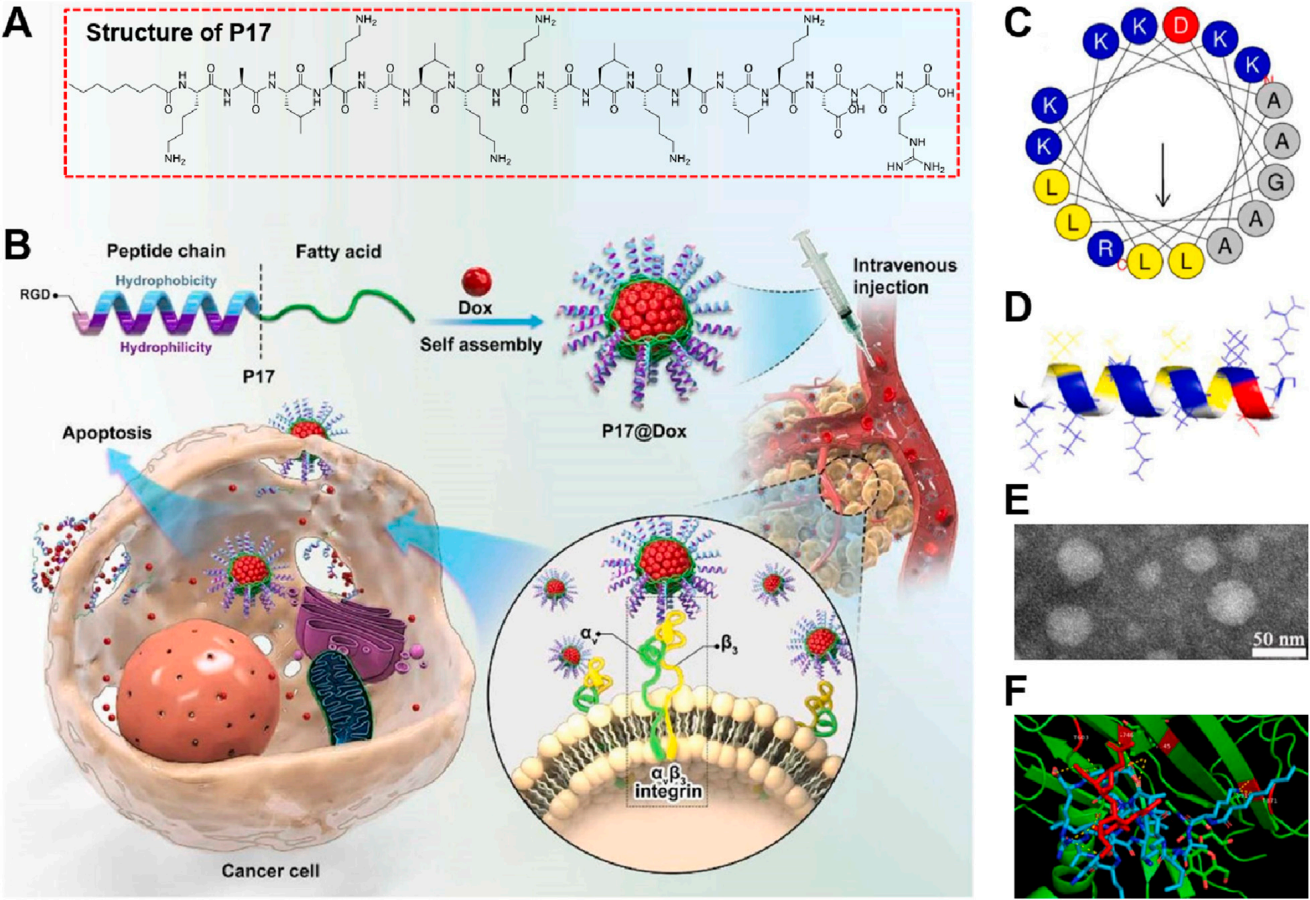
**(A)** Structure of P17 lipopeptide, showing the integration of RGD and KLA peptides modified with n-octanoic acid for improved stability and membrane binding capacity. **(B)** Schematic representation of P17 lipopeptide self-assembly and its encapsulation of doxorubicin (Dox) to form P17@Dox nanomedicine. The nanomedicine targets αvβ3 integrin on cancer cells, facilitating drug delivery and inducing apoptosis. **(C)** α-Helical projection of the P17 lipopeptide, indicating its amphiphilic nature with distinct hydrophilic and hydrophobic sides. **(D)** 3D structure of P17, highlighting its α-helical configuration critical for membrane interaction. **(E)** Molecular docking pattern showing the interaction of P17 with the αvβ3 integrin receptor via multiple hydrogen bonds, enhancing tumor targeting. **(F)** TEM image of P17 aggregates, illustrating regular monodisperse spherical morphology with an average diameter of approximately 50 nm, suitable for drug delivery applications Adapted with modification from Ma et al. (2023).

Ma et al. (2023) developed and synthesized the RGD-targeted self-assembling nanodrug involved the synthesis of an amphiphilic lipopeptide named P17 (Figure 1A), which integrated the RGD peptide and KLA peptide. The RGD peptide was chosen for its ability to target the αvβ3 integrin receptor, overexpressed in tumor cells, enhancing tumor targeting and permeability. The KLA peptide was selected for its mitochondrial apoptosis-inducing properties (Figure 1B). P17 self-assembled into stable spherical aggregates in aqueous solution, capable of encapsulating anticancer drugs such as doxorubicin (Dox). The α-helical projection of P17 indicated amphiphilic properties, with one side hydrophilic and the other hydrophobic (Figure 1C). The 3D structure confirmed the α-helical formation, crucial for its membrane interaction (Figure 1D). TEM images showed regular monodisperse spheres with an average diameter of about 50 nm, supporting its suitability as a drug carrier (Figure 1E). Molecular docking patterns revealed that the RGD motif in P17 effectively interacted with the integrin αvβ3 receptor through multiple hydrogen bonds, facilitating targeted delivery and penetration into tumor cells (Figure 1F). This interaction enhanced the therapeutic efficacy of the encapsulated Dox, resulting in effective tumor growth inhibition and reduced toxic side effects. The study had several strengths in developing the KLA-RGD integrated lipopeptide for targeted cancer therapy, but also exhibited some limitations. The major drawbacks included the relatively low encapsulation efficiency of the lipopeptide for the anticancer drug Dox and the limited *in vivo* evaluation. Future research could have focused on optimizing drug loading efficiency and extending *in vivo* studies to different cancer models to better understand the therapeutic potential and safety profile of the nanomedicine.

**FIGURE 2:**
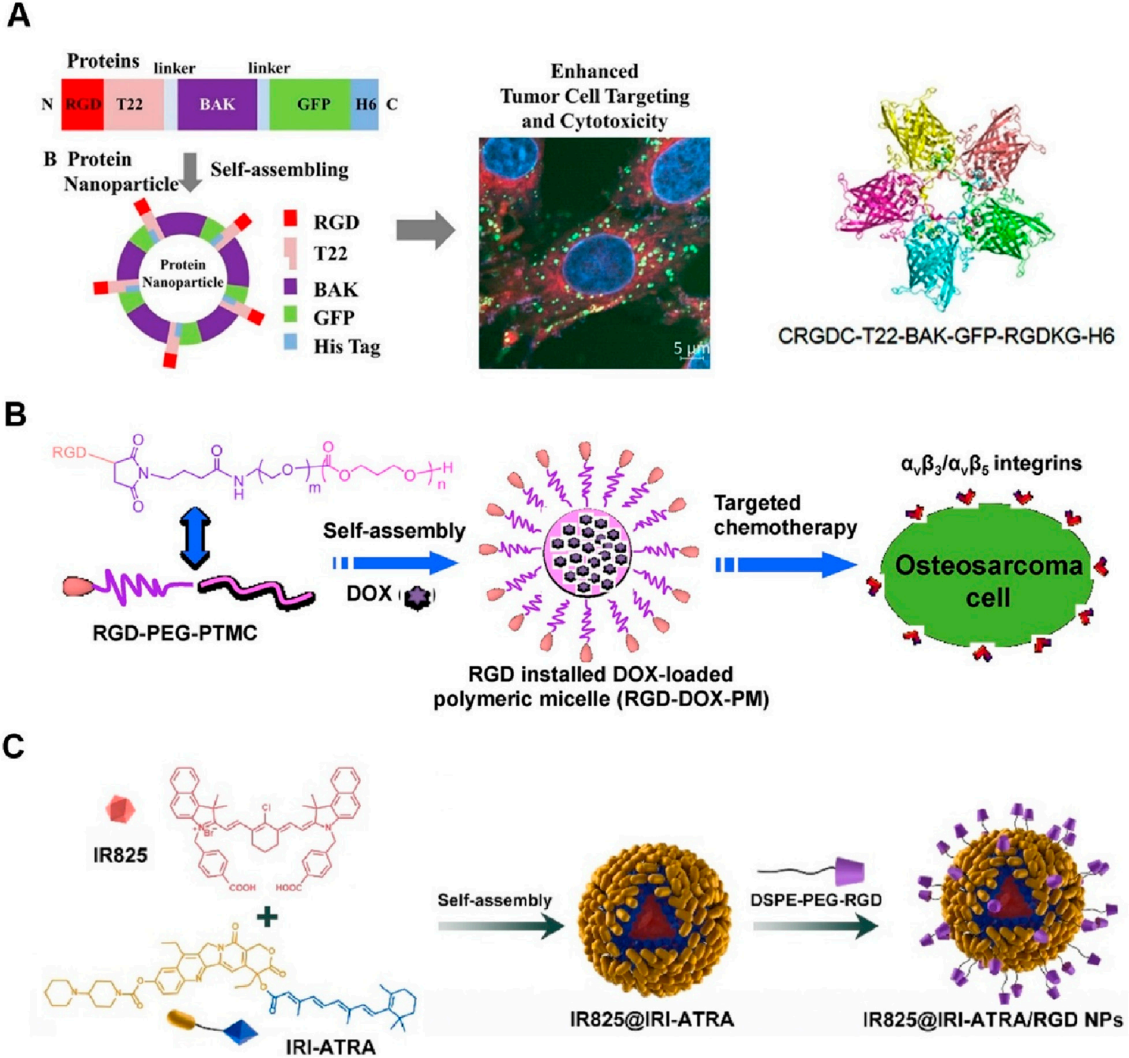
**(A)** Schematic illustration of the design and self-assembly of a protein nanoparticle incorporating RGD, T22, BAK, GFP, and His Tag for enhanced tumor cell targeting and cytotoxicity. The right panel shows the protein structure and a confocal microscopy image demonstrating targeted internalization in tumor cells Adapted with modification from Lv et al. (2020). **(B)** Diagram of the RGD-installed DOX-loaded polymeric micelle (RGD-DOX-PM) for targeted osteosarcoma chemotherapy. The RGD-PEG-PTMC micelle self-assembles and targets αvβ3/αvβ5 integrins on osteosarcoma cells, facilitating enhanced drug delivery Adapted with modification from Fang et al. (2017). **(C)** Formation process of IR825@IRI-ATRA/RGD NPs. IR825 and IRI-ATRA self-assemble into nanoparticles, which are then modified with DSPE-PEG-RGD to enhance tumor targeting and imaging capabilities Adapted with modification from Xie et al. (2022).

In Zheng’s design, the recombinant proteins, incorporating RGD peptides for targeting integrin αvβ3, BAK for pro-apoptotic activity, GFP for tracking, and a histidine tag for purification, were engineered to self-assemble into protein nanoparticles (Lv et al., 2020). These nanoparticles demonstrated a uniform size of approximately 23 nm and stability in human serum. *In vitro* studies revealed that these nanoparticles exhibited significantly enhanced cellular uptake and cytotoxicity in tumor cell lines, including C6, C26, and MCF-7 cells, compared to nanoparticles without the RGD peptide (Figure 2A). Confocal microscopy confirmed the targeted internalization of the nanoparticles in tumor cells, indicating the effectiveness of the RGD peptide in enhancing tumor targeting. This research underscores the potential of RGD-based self-assembling protein nanoparticles for targeted cancer therapy by improving delivery and therapeutic efficacy. Notably, this method could be improved by conducting *in vivo* experiments to confirm the efficacy and safety of the RGD-based self-assembling protein nanoparticles in animal models.

Fang et al. (2017) have reported the design and development of a novel DOX-loaded RGD-terminated poly(ethylene glycol)-block-poly(trimethylene carbonate) (RGD-PEG-PTMC) polymeric micelle for targeted osteosarcoma chemotherapy. These RGD-installed micelles demonstrated enhanced tumor targeting and cellular uptake in osteosarcoma cells, with significantly lower IC_50_ values compared to non-targeted micelles (Figure 2B). The micelles showed efficient drug loading and release properties, with *in vitro* studies confirming their potential for improved and targeted osteosarcoma treatment. However, further *in vivo* studies are necessary to confirm their efficacy and safety in animal models. Similarly, Zhang and coworkers developed linear RGD-based self-assembling nanodrugs that enhanced tumor targeting and cytotoxicity *in vitro*, and Song’s group created amphiphilic camptothecin nanoparticles with glutathione-responsive and tumor-targeting abilities, demonstrating prolonged circulation and improved colorectal cancer therapy (Shi et al., 2019; Song et al., 2023). However, both studies underscored the need for further *in vivo* validation.

The study by Xie et al. (2022) reported the development of a self-assembled nanodrug composed of IR825, IRI-ATRA, and DSPE-PEG-RGD for the combination therapy of breast cancer stem cells. The nanodrug (IR825@IRI-ATRA/RGD NPs) exhibited self-assembly into nanoparticles, which demonstrated excellent tumor targeting and imaging capabilities. Upon cellular uptake, the nanoparticles effectively released their therapeutic agents in response to the acidic and esterase-rich tumor microenvironment (Figure 2C). The study highlighted significant cytotoxicity, enhanced cellular uptake, and superior photothermal properties, resulting in effective tumor growth inhibition and metastasis prevention. *In vivo* studies confirmed the potential of this nanodrug for improved breast cancer therapy. The study could be improved by conducting more extensive *in vivo* experiments to evaluate long-term safety and efficacy, as well as exploring the potential for scaling up the synthesis process for clinical applications. Jia utilized ffKGRGD, a chain RGD peptide, as a tumor-targeting ligand to enhance the therapeutic efficacy of self-assembling nanodrugs. The ffKGRGD-modified nanodrugs significantly improved tumor targeting, cellular uptake, and antitumor activity in cancer models by promoting integrin receptor-mediated endocytosis (Jia et al., 2022).

### 3.2 Cyclo(RGDfC)-based self-assembling nanodrugs

Cyclo(RGDfC)-based self-assembling nanodrugs are innovative therapeutic agents for cancer treatment. The cyclic RGDfC peptides, stabilized by disulfide bonds, form stable nanostructures, enhancing drug delivery efficiency. These nanodrugs self-assemble into nanoparticles, encapsulating chemotherapeutic agents for controlled release, thus increasing drug concentration at the tumor site while minimizing systemic toxicity. Future research should optimize their stability, biocompatibility, and scalability for clinical applications.

**FIGURE 3:**
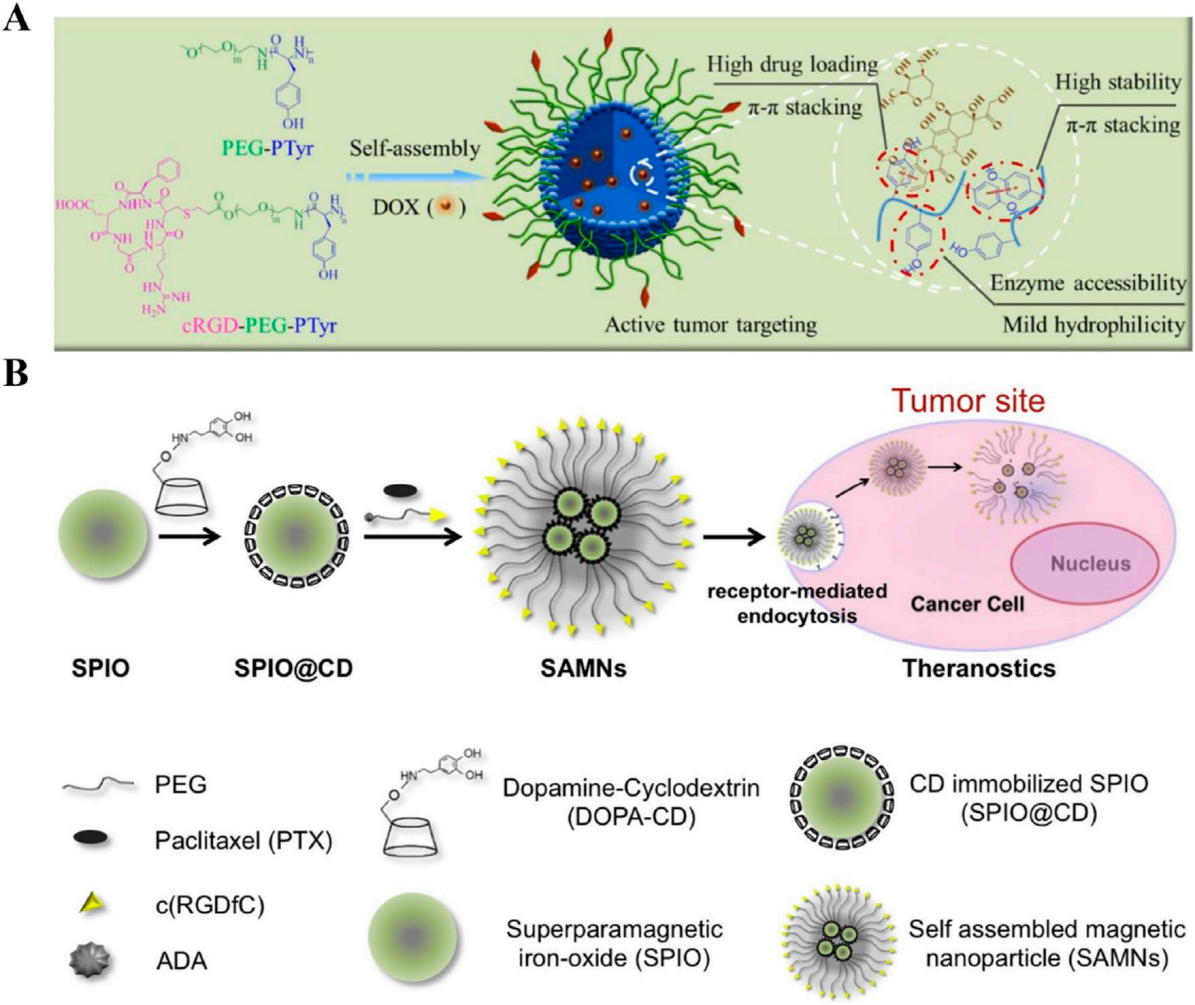
**(A)** Schematic of the formation of cyclo(RGDfC)-based self-assembling nanodrugs. Cyclo(RGDfC) peptides form stable nanoparticles through disulfide bonds, encapsulating chemotherapeutic agents like doxorubicin (DOX) for enhanced drug delivery Adapted with modification from Gu et al. (2019). **(B)** Illustration of the preparation of magnetic nanoclusters (SAMNs) coated with β-cyclodextrin (CD) and polyethylene glycol (PEG), followed by surface functionalization with cyclo(RGDfC). This functionalization improves cellular uptake, targeted delivery to integrin-rich cancer cells, and controlled release of paclitaxel (PTX), leading to better MRI contrast and therapeutic outcomes Adapted with modification from Nguyen et al. (2016).

Gu et al. (2019) have developed the cyclo(RGDfC)-based self-assembling nanodrugs for targeted cancer therapy. Cyclo(RGDfC) peptides, stabilized by disulfide bonds, form stable nanoparticles that enhanced drug delivery. These nanoparticles targeted αvβ3 and αvβ5 integrin receptors overexpressed on tumor cells, ensuring specific drug delivery. The amphiphilic peptides facilitated stable self-assembly and controlled release of doxorubicin (DOX) in the tumor microenvironment, increasing drug concentration at the tumor site while minimizing systemic toxicity (Figure 3A). *In vitro* and *in vivo* experiments demonstrated superior antitumor activity, higher cellular uptake, and improved tumor targeting compared to non-targeted formulations. Despite promising results, challenges like optimizing biocompatibility, stability, and scalability need to be addressed for clinical translation. Such Cyclo(RGDfC)-based self-assembling nanodrugs have also been witnessed in several other studies (Liang et al., 2016; He et al., 2016; Li et al., 2019).

Alternatively, Nguyen et al. (2016) have developed self-assembling nanodrugs where Cyclo(RGDfC) was attached to the surface of pre-formed nanoparticles. Superparamagnetic iron oxide nanoparticles (SPIO) were first coated with β-cyclodextrin (CD) and polyethylene glycol (PEG) to form stable, self-assembling magnetic nanoclusters (SAMNs). The SAMNs were further functionalized with the tumor-targeting peptide Cyclo(RGDfC) (Figure 3B). This modification enhanced the uptake of nanodrugs into tumor cells by targeting integrin receptors, which are overexpressed on cancer cells. The nanoclusters showed improved drug loading efficiency and controlled release of paclitaxel (PTX), leading to enhanced magnetic resonance imaging (MRI) contrast and targeted drug delivery in cancer therapy. *In vitro* studies demonstrated that the Cyclo(RGDfC)-functionalized SAMNs had superior cellular uptake and higher antitumor efficacy compared to non-targeted formulations. The SAMNs provided a controlled release of PTX, which was triggered by competitive guest molecules, ensuring efficient drug delivery to tumor sites while minimizing systemic toxicity. These findings suggest that Cyclo(RGDfC)-functionalized SAMNs have significant potential for application in image-guided cancer chemotherapy. Notably, this study needs improvements in biocompatibility, scalability, drug loading and release optimization, targeting efficiency, and comprehensive *in vivo* efficacy studies to facilitate clinical translation. Additionally, aligning with regulatory guidelines is crucial for future development. Such strategy for the development of Cyclo(RGDfC)-based nanodrugs have also been utilized in Ding, Zhang, and Bao’s study, respectively (Ding et al., 2021; Zhang et al., 2024; Bao et al., 2016). Ding et al. (2021)’s study demonstrated that CDDP-loaded polyamino acid nanoparticles, combined with RGD peptides, improved tumor targeting and reduced toxicity in non-small cell lung cancer models. Zhang et al. (2024)’s research focused on zwitterionic dendrimer self-assembled nanodrugs, highlighting their high drug loading and enhanced anti-tumor efficacy in acidic tumor environments. Bao et al. (2016)’s work illustrated the superior tumor targeting and antitumor activity of cRGD-modified micelles loaded with DOX, showcasing their potential in overcoming multidrug resistance.

### 3.3 Cyclo(RGDfK)-based self-assembling nanodrugs

Cyclo(RGDfK) is a cyclic peptide known for its high binding affinity to integrin receptors overexpressed in tumor cells. Its stability and specificity make it particularly effective for targeted drug delivery in cancer therapy. Cyclo(RGDfK)-based self-assembling nanodrugs are designed for targeted cancer therapy, enhancing tumor targeting, drug delivery efficiency, and therapeutic outcomes by leveraging integrin receptor interactions.

**FIGURE 4:**
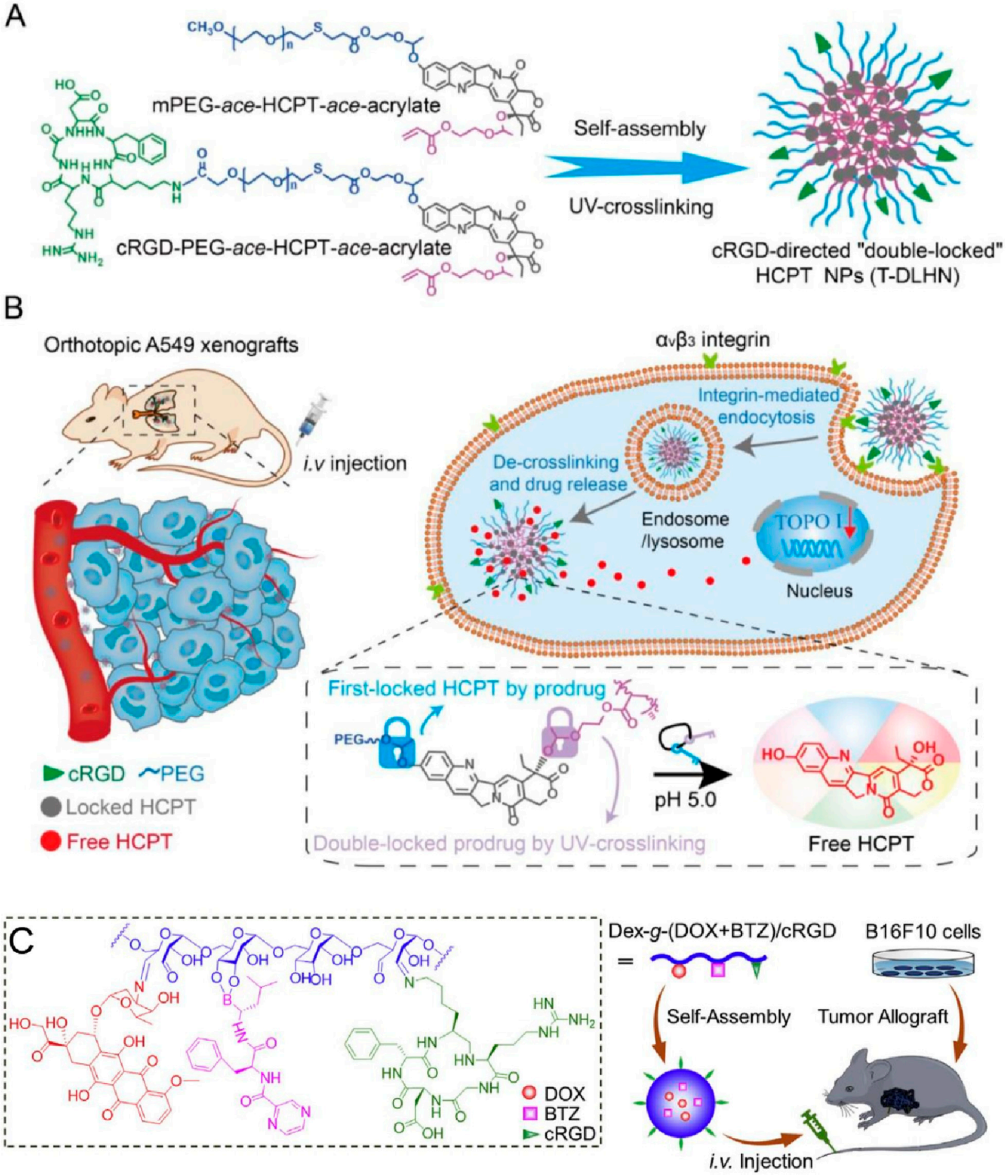
**(A)** Confocal images of T-24 cells with FITC-labeled micelles; left panel: non-modified micelles, right panel: RGD-modified micelles, showing higher uptake for RGD-modified micelles. **(B)** Flow cytometry analysis indicating increased fluorescence intensity in T-24 cells treated with RGD-modified micelles, confirming enhanced targeting Adapted with modification from Zhang J. et al. (2023). **(C)** Quantitative uptake analysis over time, with RGD-modified micelles showing higher fluorescence at all-time points compared to non-modified micelles Adapted with modification from Li et al. (2020).

Zhong’s group developed cyclo (RGDfK)-based self-assembling nanodrugs for targeted lung cancer therapy, focusing on a “double-lock” mechanism for enhanced stability and efficacy (Zhang J. et al., 2023). The nanodrugs were prepared by first assembling HCPT-loaded nanoparticles using mPEG-ace-HCPT-ace-acrylate and cRGD-PEG-ace-HCPT-ace-acrylate, followed by UV-crosslinking to create the stable “double-locked” nanostructures (T-DLHN) (Figure 4A). Cyclo (RGDfK) peptides on the surface of these nanoparticles facilitated targeted delivery to tumor cells. Figure 4B illustrates the therapeutic mechanism in orthotopic A549 lung cancer xenografts. The cRGD-functionalized nanoparticles targeted αvβ3 integrin receptors on tumor cells, enhancing cellular uptake through integrin-mediated endocytosis. Once internalized, the nanoparticles underwent de-crosslinking and drug release in the acidic tumor microenvironment, effectively delivering HCPT to the nucleus, inhibiting topoisomerase I, and inducing cell death. *In vivo* studies demonstrated that these nanodrugs significantly prolonged circulation time, improved tumor accumulation, and enhanced antitumor efficacy while minimizing systemic toxicity, making them a promising strategy for safe and efficient chemotherapy in lung cancer. This method needs improvements in optimizing the biocompatibility and long-term safety of the nanodrugs, particularly in terms of potential immune responses. Additionally, further research is required to enhance the scalability and cost-effectiveness of the production process for clinical applications.

In another study, the authors developed a Cyclo (RGDfK)-based self-assembling nanodrug, where HCPT was conjugated with cRGD-PEG and self-assembled into nanoparticles (Li et al., 2020). The resulting “double-locked” nanoparticles (T-DLHN) exhibited enhanced stability and tumor-targeting capabilities. The nanodrug demonstrated effective integrin-mediated endocytosis and acid-triggered drug release, leading to significant tumor suppression in orthotopic A549 xenograft models with minimal systemic toxicity (Figure 4C). The method requires improvements in optimizing the drug release rate and ensuring the long-term stability of the nanoparticles in the physiological environment. Further studies are needed to evaluate the safety and efficacy of these nanodrugs in clinical settings. Additionally, Cyclo(RGDfK) have also been utilized for the synthesize of several other self-assembling nanodrugs for cancer therapy. Chen’s study described a novel strategy to preserve structurally labile peptide assemblies after molecular modification by designing sheet-forming peptides that allow for staggered alignment, enabling the creation of densely functionalized nanosheets with various molecules, including c(RGDfK) (Chen et al., 2024). This study faced challenges in maintaining the structural integrity of peptide assemblies after molecular functionalization, requiring improved methods to preserve assembly morphology while achieving high functionalization density. Zhou’s study developed a targeted drug delivery system using c(RGDfK)-decorated micelles for intravesical instillation chemotherapy of superficial bladder cancer, demonstrating high affinity to bladder cancer cells and significant inhibitory effects on cell proliferation (Nguyen et al., 2016). In addition to improving stability, the c(RGDfK) modification was shown to enhance the biodistribution of the micelles by promoting selective accumulation at the tumor site through specific binding to integrin receptors, thereby reducing off-target effects and systemic toxicity. This selective targeting and improved biodistribution highlight the potential of c (RGDfK)-decorated micelles to optimize drug delivery efficiency. However, the study identified the need to further enhance the stability and drug loading efficiency of c(RGDfK)-decorated micelles for effective bladder cancer treatment, suggesting further optimization of the micelle formulation (Zhou et al., 2013). Zou et al. (2016) reported on self-crosslinkable and intracellularly decrosslinkable micellar nanoparticles decorated with c (RGDfK), showing enhanced targeted delivery and therapeutic efficacy against melanoma and glioma cells, highlighting their potential for clinical translation. This research highlighted the complexity and potential safety concerns of self-crosslinkable micellar nanoparticles, indicating a need for simpler and safer design for clinical translation.

### 3.4 Cyclo(RGDyK)-based self-assembling nanodrugs

Cyclo(RGDyK)-based self-assembling nanodrugs offer a promising approach for targeted cancer therapy by leveraging the specific binding affinity of the cyclic peptide Cyclo(RGDyK) to integrin receptors overexpressed on tumor cells. These nanodrugs self-assemble into stable nanoparticles that encapsulate chemotherapeutic agents, ensuring controlled release and enhanced drug concentration at the tumor site. Cyclo(RGDyK) has a high binding affinity for integrin receptors, particularly αvβ3 and αvβ5, which are overexpressed on tumor cells, enhancing specificity and targeting efficiency. Such targeted delivery system minimizes systemic toxicity and improves therapeutic efficacy. Future research should focus on optimizing the stability, biocompatibility, and clinical scalability of these nanodrugs for effective cancer treatment.

**FIGURE 5:**
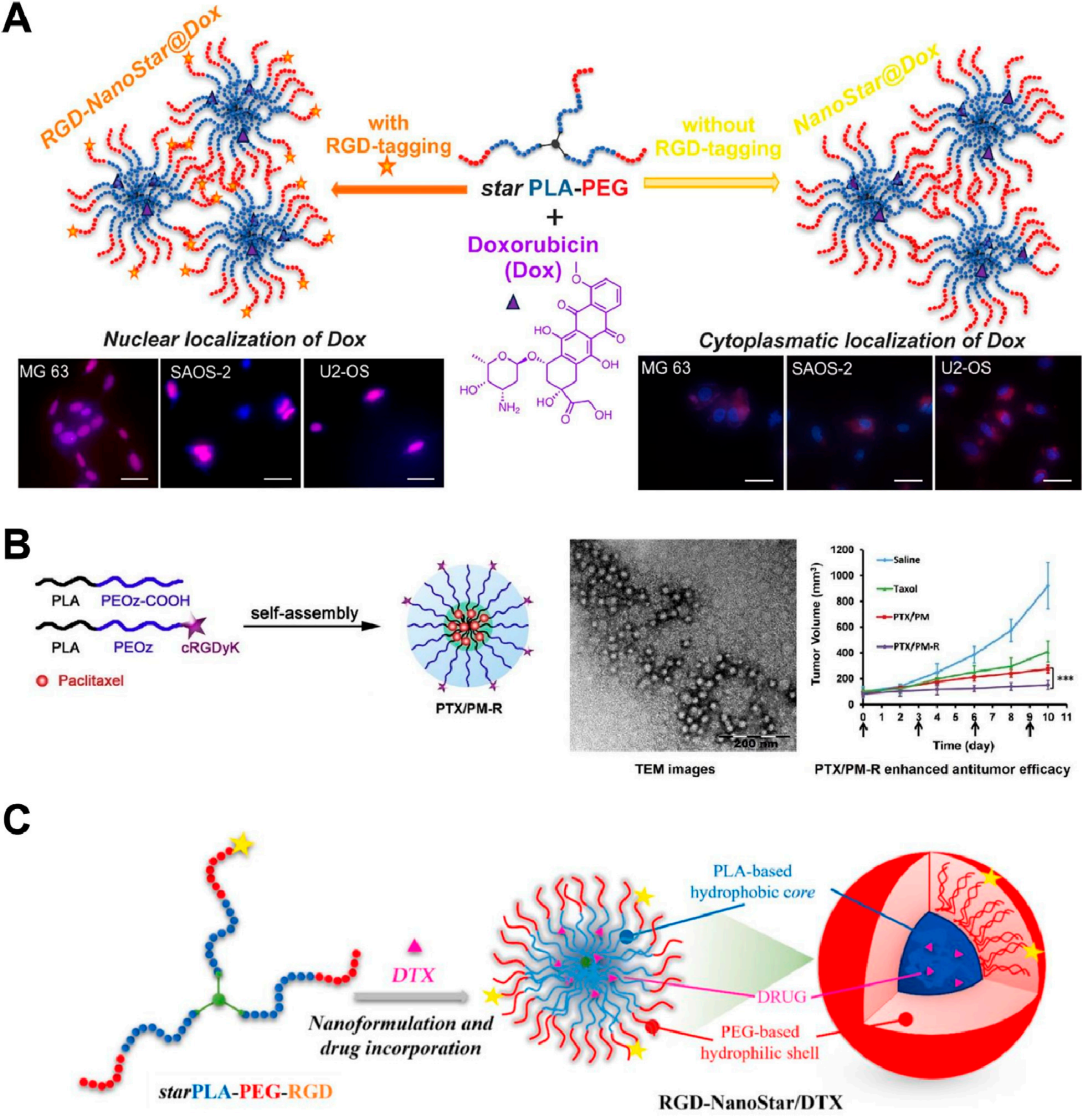
**(A)** Schematic and fluorescence images of RGD-tagged (RGD-NanoStar@Dox) versus non-tagged (NanoStar@Dox) micellar nanoassemblies for Dox delivery. RGD-tagged nanoassemblies showed enhanced nuclear localization of Dox in osteosarcoma cells (MG63, SAOS-2, U2-OS), indicating effective integrin-mediated endocytosis Adapted with modification from Oliva et al. (2024). **(B)** Schematic and TEM images of cyclic RGDyK-conjugated, paclitaxel-loaded pH-responsive polymeric micelles (PTX/PM-R). TEM images confirmed uniform spherical nanoparticles. *In vivo*, PTX/PM-R significantly suppressed tumor growth compared to PTX/PM and Taxol, due to improved tumor targeting and pH-responsive drug release Adapted with modification from Gao et al. (2015). **(C)** Schematic, TEM images, and tumor volume reduction graph of star-shaped PLA-PEG-RGD nanoshuttles for docetaxel (DTX) delivery. TEM confirmed nanoparticle structure. *In vivo*, RGD-NanoStar/DTX significantly reduced tumor volume compared to saline, Taxol, and untargeted formulations, due to enhanced targeting and uptake by tumor cells Adapted with modification from Torcasio et al. (2022).

Oliva et al. (2024) developed star-shaped PLA-PEG micellar nanoassemblies tagged with cyclic RGDyK (RGD-NanoStar@Dox) for targeted delivery of doxorubicin (Dox) to osteosarcoma cells. The RGD-tagged nanoassemblies demonstrated enhanced nuclear localization of Dox in MG63, SAOS-2, and U2-OS osteosarcoma cells, as evidenced by the fluorescence images showing Dox accumulation in the cell nuclei (Figure 5A). In contrast, untargeted NanoStar@Dox showed cytoplasmic localization of Dox, indicating ineffective delivery to the nucleus. This highlights the crucial role of the cyclic RGDyK peptide in facilitating integrin-mediated endocytosis and efficient drug delivery to the tumor cell nucleus, enhancing therapeutic efficacy while minimizing effects on healthy osteoblasts (hFOBs). Notably, one limitation of this method is the potential for immune response due to the presence of RGD peptides, and there is a need to further investigate long-term safety and efficacy *in vivo*. Additionally, optimizing the drug release rate to ensure a more consistent therapeutic effect is necessary.

In another study, researchers developed cyclic RGDyK-conjugated, paclitaxel-loaded pH-responsive polymeric micelles (PTX/PM-R) for targeted cancer therapy (Gao et al., 2015). The micelles self-assembled into nanoparticles, enhancing drug delivery and tumor targeting efficiency. Transmission electron microscopy (TEM) images confirmed the uniform spherical structure of these nanoparticles. *In vivo* studies demonstrated that PTX/PM-R significantly suppressed tumor growth in comparison to non-targeted micelles (PTX/PM) and Taxol (Figure 5B). The enhanced antitumor efficacy was attributed to the improved targeting and uptake by tumor cells, facilitated by the cyclic RGDyK peptide, and the pH-responsive release of paclitaxel within the tumor microenvironment. However, one potential drawback of this method is the complexity of synthesizing and optimizing the pH-responsive polymeric micelles, which may impact large-scale production. Additionally, further investigation into the long-term stability and potential immune responses *in vivo* is necessary to ensure safety and efficacy.

Torcasio et al. (2022) developed star-shaped PLA-PEG-RGD nanoshuttles for docetaxel (DTX) delivery, forming self-assembled micelles with a hydrophobic core and hydrophilic shell. TEM confirmed the nanoparticle structure. *In vivo* tests demonstrated that RGD-NanoStar/DTX significantly reduced tumor volume compared to saline, Taxol, and untargeted formulations. The improved efficacy was attributed to the enhanced targeting and uptake by tumor cells, facilitated by the RGD peptide, leading to better drug accumulation and antitumor effects (Figure 5C). This method needs to further optimize drug loading efficiency and stability of the nanoshuttles to ensure consistent therapeutic outcomes. Such design has also been also applied in Dai and Fang’s study. Dai’s study has demonstrated that cyclic RGDyK-conjugated nanoparticles significantly enhanced the penetration and chemotherapy efficacy of paclitaxel against advanced gliomas. These nanoparticles showed improved tumor targeting, deeper tissue penetration, and higher accumulation in glioma cells, leading to prolonged survival in mice models (Wang et al., 2021). Fang and coworkers have developed cyclic RGDyK-functionalized poly(trimethylene carbonate)-based nanoparticles for paclitaxel delivery. The results showed enhanced endocytic uptake by glioma cells, improved tumor penetration, and superior antitumor effects compared to non-targeted nanoparticles and conventional treatments, with minimal *in vivo* toxicity (Jiang et al., 2013).

### 3.5 Cyclo(RGDyC)-based self-assembling nanodrugs

Cyclo(RGDyC) has a unique cysteine (C) residue that can form a disulfide bond, which significantly enhances their stability and resistance to enzymatic degradation. The presence of the cysteine residue allows for site-specific conjugation with various therapeutic agents or imaging molecules, facilitating multifunctional applications. This feature makes Cyclo(RGDyC) particularly suitable for developing targeted therapies that require stable, long-circulating nanocarriers with the ability to deliver payloads precisely to tumor sites. Additionally, Cyclo(RGDyC) exhibits a distinct affinity for certain integrin subtypes, making it particularly effective in targeting tumors with specific integrin expressions, such as gliomas and metastatic cancers, where stability and precise targeting are crucial.

**FIGURE 6:**
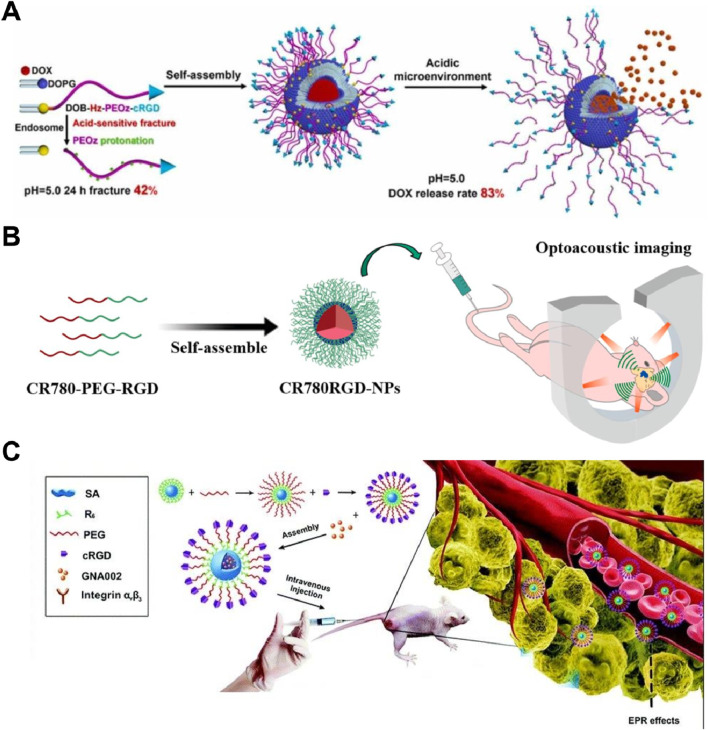
**(A)** cPzDGX liposomes with Cyclo (RGDyC) showed enhanced DOX release and uptake in A549/ADR cells, reducing cell viability and 3D tumor spheroid diameter Adapted with modification from Chen et al. (2023). **(B)** CR780RGD-NPs, utilizing Cyclo (RGDyC), demonstrated superior optoacoustic imaging efficiency and specificity for brain tumors, with significant signal retention at 5 mm depth Adapted with modification from Liu N. et al. (2021). **(C)** cPHRS nanocarriers with Cyclo (RGDyC) facilitated targeted drug delivery, showing increased cytotoxicity against cancer cells and notable tumor growth inhibition *in vivo* Adapted with modification from Li et al. (2021).

The cRGD-PEOz-Hz-DOB molecules, incorporating Cyclo(RGDyC), facilitated specific binding to αvβ3 receptors on tumor cells, enhancing targeted delivery and cellular uptake (Chen et al., 2023). At pH 5.0, the hydrazone bond in cPzD exhibited a 42% breakage rate within 48 h, resulting in an 82.9% cumulative release rate of DOX in acidic conditions, which was 22.5% higher than at pH 7.4. Uptake of cPzDGX in A549/ADR cells increased by 62% at 2 h, and EGFP expression was 36% higher compared to the control group, indicating that Cyclo(RGDyC) significantly improved the liposomes’ targeting ability (Figure 6A). Additionally, the cell viability of the cPzDGX group decreased by 17.2% relative to the free DOX group, and the diameter reduction of 3D tumor microspheres increased by 34.4%, demonstrating enhanced anticancer efficacy due to Cyclo (RGDyC). The study’s main limitation is the lack of *in vivo* testing to confirm the therapeutic efficacy and safety of cPzDGX liposomes, as well as potential immune responses over prolonged use. Future research should focus on optimizing the stability of these liposomes in biological fluids and improving large-scale manufacturing processes to ensure consistent quality and effectiveness.

Liu N. et al. (2021) demonstrated that CR780RGD-NPs enabled efficient optoacoustic imaging of brain tumors with high specificity and strong signal generation, exhibiting a 1.85 times higher optoacoustic generation efficiency compared to ICG. These nanoparticles retained a strong signal at a depth of 5 mm in brain tissue and successfully penetrated the blood-brain barrier, showing a 3.5-fold increase in optoacoustic intensity in tumor regions (Figure 6B). CR780RGD-NPs also showed effective targeting and retention in brain tumors for at least 24 h, confirming their potential for precise tumor imaging and diagnosis. A unique limitation of this study was the insufficient exploration of the long-term *in vivo* stability of CR780RGD-NPs in the brain tumor microenvironment. Li et al. (2021) designed a pH-sensitive nanocarrier, cRGD-PEG-Hyd-R6-SA (cPHRS), utilizing Cyclo(RGDyC) for enhanced tumor targeting. Cyclo(RGDyC) facilitated specific binding to αvβ3 integrins on cancer cells, improving drug accumulation via receptor-mediated endocytosis. The average size of drug-loaded nanoparticles was 143.13 nm, and they exhibited stability in physiological conditions but disassembled in acidic environments, releasing GNA002 (Figure 6C). This system significantly enhanced cytotoxicity against HN6, HeLa, and A549 cells and demonstrated notable tumor growth inhibition *in vivo*, confirming its potential for precise and efficient cancer therapy. Notably, the long-term stability of these nanocarriers in the acidic tumor microenvironment is unknown. Ouyang et al. developed cRGD-PAE-PEG-DSPE nanoparticles for targeted docetaxel delivery, enhancing tumor targeting via Cyclo(RGDyC). These nanoparticles demonstrated high serum stability and acid responsiveness, leading to increased uptake by MDA-MB-231 cells and significant tumor growth inhibition *in vivo* compared to free docetaxel, confirming their potential for efficient and targeted cancer therapy (Ouyang et al., 2024).

### 3.6 iRGD-based self-assembling nanodrugs

**FIGURE 7:**
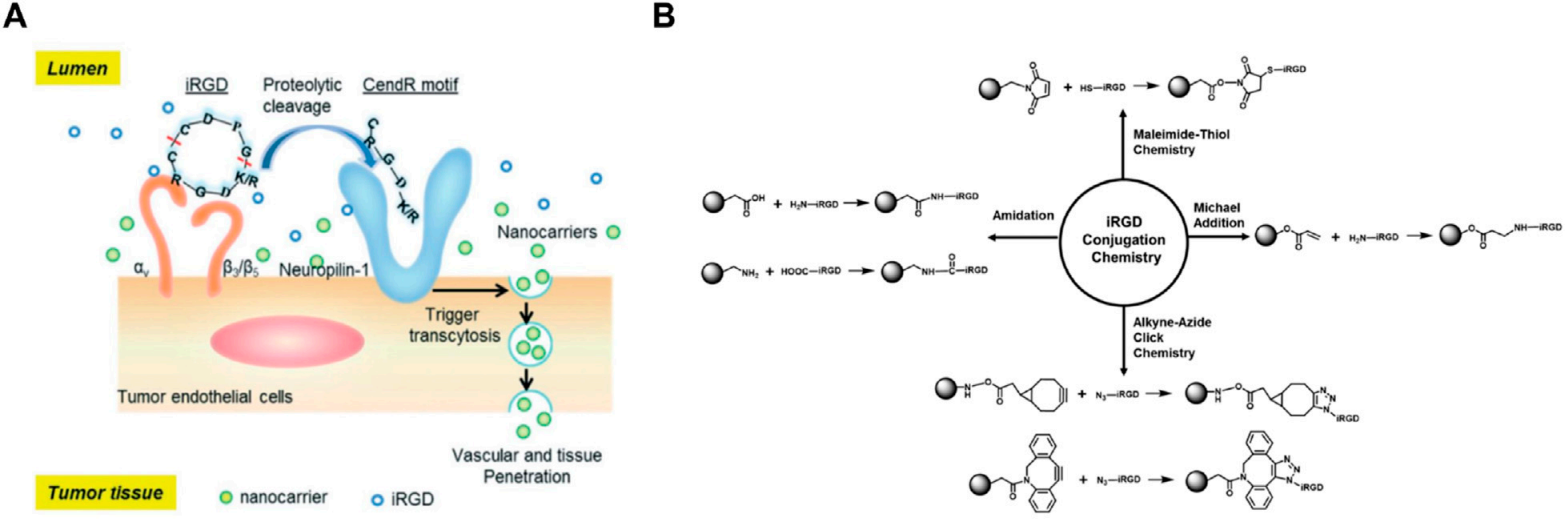
**(A)** Schematic representation of the iRGD peptide mechanism, showing binding to αvβ3 integrins on tumor endothelial cells, proteolytic cleavage, and subsequent interaction with NRP-1 receptors to facilitate nanocarrier penetration into tumor tissue. **(B)** Illustration of various bioconjugation reactions used for attaching iRGD to nanocarriers, including maleimide-thiol, Michael addition, azide-alkyne click chemistry, and amidation reactions, enhancing targeted drug delivery to tumors Adapted with modification from Liu et al. (2017).

iRGD (internalizing RGD) is a tumor-penetrating peptide that combines the RGD motif for integrin binding with a CendR motif (sequence CRGDRGPDC) that activates a tissue-penetrating pathway. This dual functionality makes iRGD particularly effective for tumor targeting and penetration. The RGD motif in iRGD binds to αvβ3 and αvβ5 integrins, which are overexpressed on tumor cells and their vasculature (Liu et al., 2017; Sugahara et al., 2010). Upon binding, iRGD undergoes proteolytic cleavage to expose the CendR motif, which interacts with neuropilin-1 (NRP-1) receptors, facilitating deep tissue penetration and enhanced drug delivery into the tumor parenchyma (Figure 7A). This unique mechanism allows iRGD-based self-assembling nanodrugs to not only target tumor cells with high specificity but also penetrate deeply into tumor tissues, overcoming the limitations of poor drug distribution within solid tumors. Additionally, iRGD can enhance the permeability of tumor vasculature, improving the delivery of co-administered therapeutic agents (Figure 7B). These properties make iRGD-based nanodrugs highly promising for improving the efficacy of cancer treatments by ensuring that therapeutic agents reach all areas of the tumor, including those that are difficult to access with conventional drugs. The ability of iRGD to enhance both targeting and penetration significantly increases the therapeutic index of the encapsulated drugs, making it a valuable component in the design of advanced nanodrug delivery systems (Pang et al., 2014; Simón-Gracia et al., 2016).

Unlike other cyclic RGD peptides, iRGD is a tumor-penetrating peptide that combines cell-homing and cell-penetrating abilities. iRGD specifically binds to integrin receptors, particularly αvβ3 and αvβ5, which are overexpressed on tumor vasculature. Upon binding, iRGD undergoes proteolytic cleavage, exposing the CendR motif (C-end rule), which activates neuropilin-1 (NRP-1) receptors on tumor cells. This interaction triggers tissue penetration, facilitating the delivery of therapeutic agents deep into the tumor parenchyma. As a result, iRGD enhances both the targeting and penetration of drug-loaded nanoparticles, significantly improving their therapeutic index and distribution within solid tumors (Sugahara et al., 2010; Teesalu et al., 2013; Jiang et al., 2022).

**FIGURE 8:**
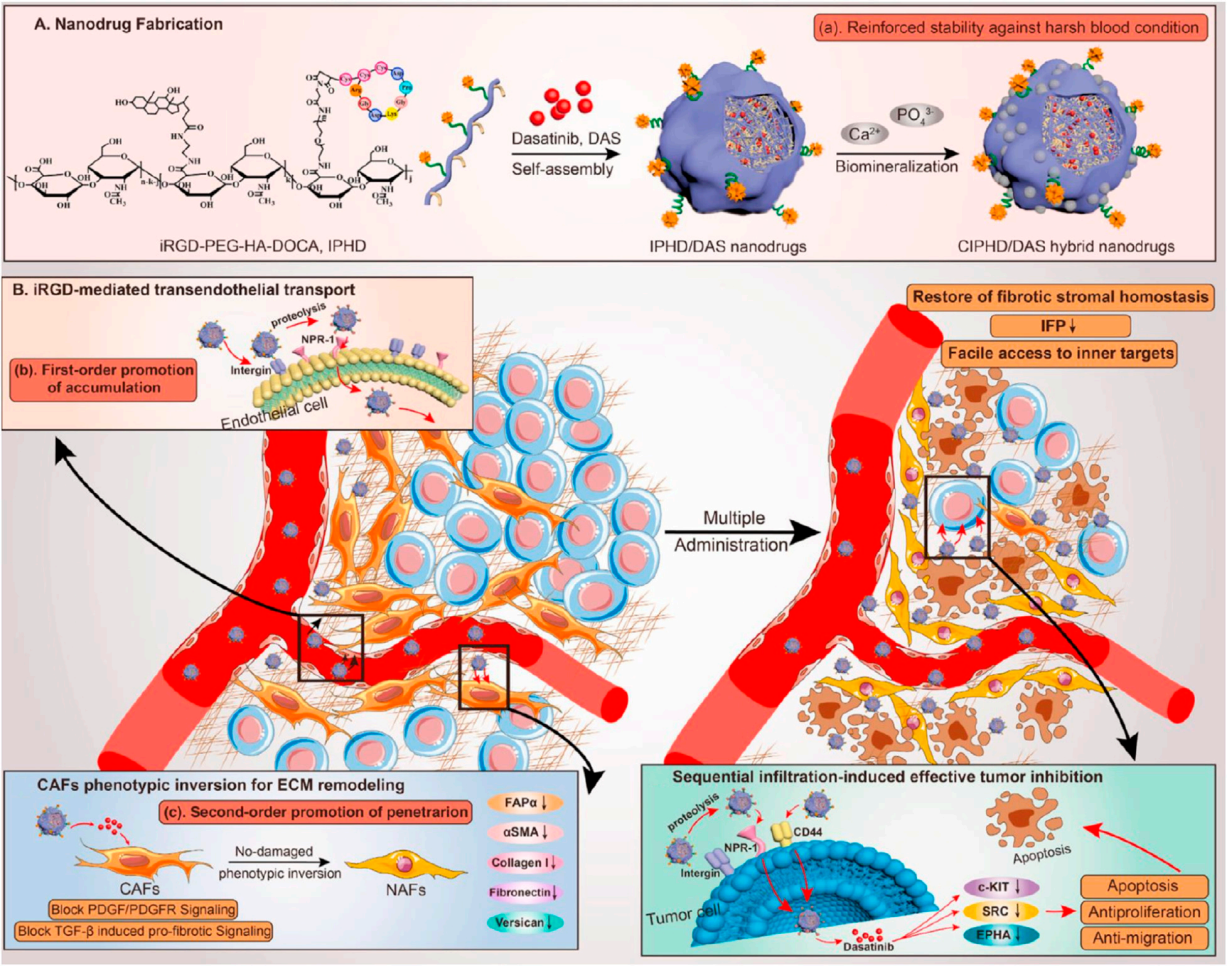
**(A)** iRGD-based self-assembling nanodrug fabrication, illustrating the synthesis and assembly process of iRGD-PEG-HA-DOCA and CIPHD/DAS hybrid nanodrugs, including steps for enhanced stability in harsh blood conditions through biomineralization. **(B)** iRGD-mediated transendothelial transport and CAFs modulation, showcasing the first-order promotion of nanodrug accumulation in tumors via integrin and NRP-1 binding, and second-order promotion of penetration through ECM remodeling and CAF phenotypic inversion Adapted with modification from Liu Y. et al. (2021).

Liu Y. et al. (2021) developed iRGD-PEG-HA-DOCA and CIPHD/DAS hybrid nanodrugs for enhanced tumor targeting and penetration. These nanodrugs were fabricated through a self-assembly process followed by biomineralization to improve stability against harsh blood conditions (Figure 8A). iRGD facilitated specific binding to αvβ3 integrins and neuropilin-1 receptors on tumor endothelial cells, promoting transendothelial transport and accumulation in tumors (Figure 8B). A unique limitation this study was the insufficient evaluation of the long-term effects of CAF phenotypic reversion on overall tumor progression and recurrence. Additionally, the nanodrugs modulated cancer-associated fibroblasts (CAFs) to revert their phenotype, leading to ECM remodeling and further enhancing nanodrug penetration and tumor inhibition.

**FIGURE 9:**
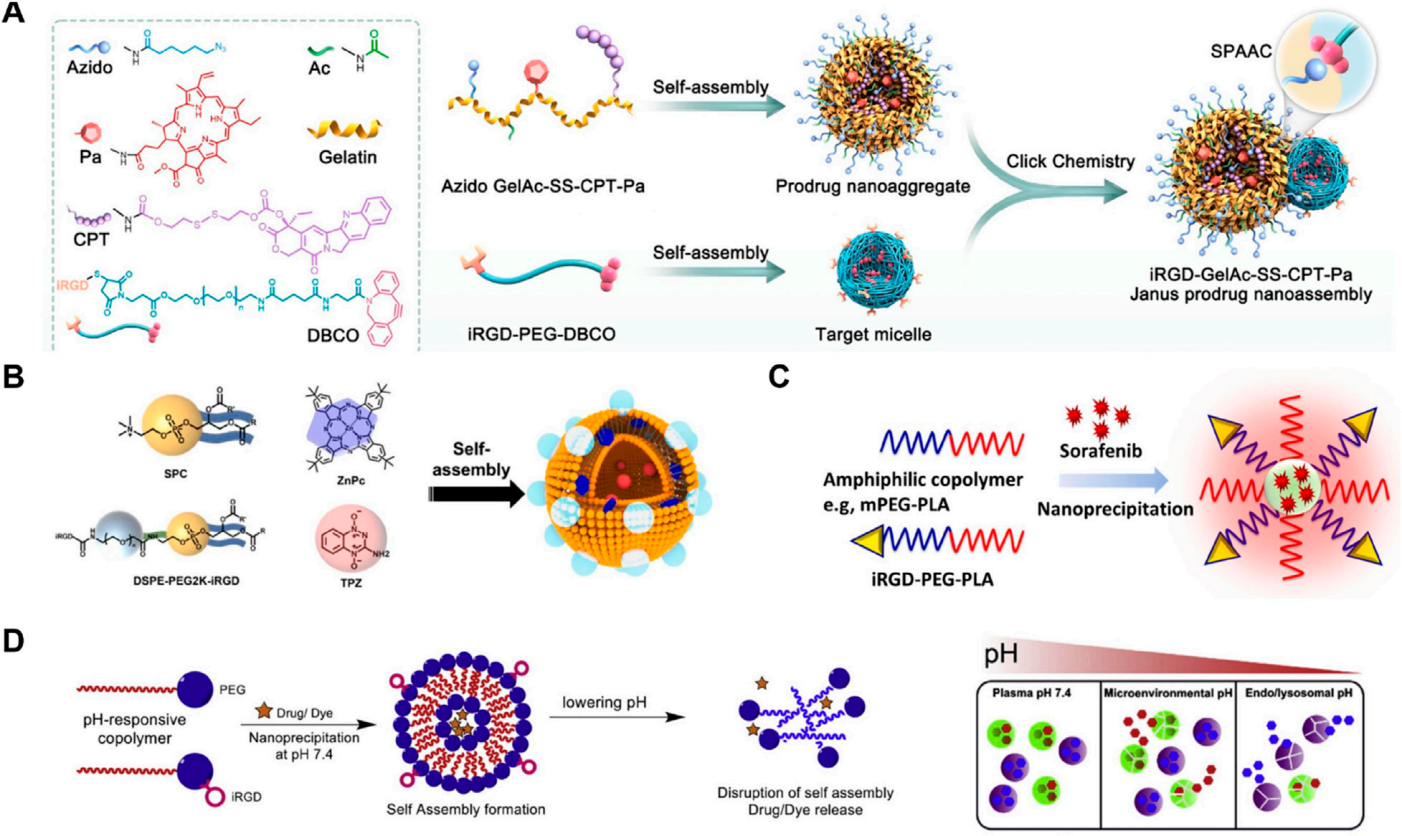
**(A)** Schematic illustration of the synthesis and self-assembly process of iRGD-GelAc-SS-CPT-Pa Janus prodrug nanoassemblies, highlighting the roles of various components and their self-assembly into nanostructures Adapted with modification from Hao et al. (2024). **(B)** *In vivo* fluorescence imaging of tumor-bearing mice post-injection with the developed nanodrugs, demonstrating the targeting and accumulation within tumor tissues over time Adapted with modification from Zhang et al. (2022). **(C)** Quantitative analysis of fluorescence intensity in *ex vivo* organs, showing significant accumulation in tumor tissues compared to other organs, indicating effective targeting and retention Adapted with modification from Hu et al. (2021). **(D)** Confocal microscopy images of tumor sections, revealing enhanced penetration and distribution of iRGD-GelAc-SS-CPT-Pa in tumor tissues, with detailed fluorescence indicating successful delivery to deeper tumor regions Adapted with modification from Ray et al. (2019).

Hao et al. (2024) developed iRGD-GelAc-SS-CPT-Pa Janus prodrug nanoassemblies, combining prodrug nanoaggregates and target micelles linked via copper-free click chemistry. The iRGD peptide enhanced tumor targeting by binding to integrins and facilitating penetration through neuropilin-1 receptors. In the tumor microenvironment, the high concentration of MMP2 triggered the degradation of the prodrug nanoaggregates into smaller nanoparticles, improving cellular uptake and penetration. The prodrug released camptothecin (CPT) in response to glutathione (GSH), while photosensitizer Pa generated reactive oxygen species (ROS) under 660 nm laser irradiation, enhancing antitumor efficacy through synergistic chemotherapy and photodynamic therapy (Figure 9A). Notably, this study’s main drawback was the insufficient optimization of the gelatin-based carriers’ biocompatibility and stability under physiological conditions, necessitating improvements in material selection for enhanced performance. Zhang et al. (2022) developed iRGD@ZnPc + TPZ nanoparticles for targeted glioma therapy. The nanoparticles were self-assembled from SPC, ZnPc, DSPE-PEG2K-iRGD, and TPZ. iRGD facilitated the crossing of the blood-brain barrier and targeted gliomas, while ZnPc acted as a photosensitizer, and TPZ as a hypoxia-activated prodrug. This system enhanced tumor penetration and therapeutic efficacy through a combination of photodynamic therapy and hypoxia-activated chemotherapy (Figure 9B). Hu et al. (2021) developed iRGD-PEG-PLA nanoparticles for sorafenib delivery, enhancing tumor targeting and penetration through nanoprecipitation. The amphiphilic copolymers mPEG-PLA and iRGD-PEG-PLA facilitated the self-assembly of the nanoparticles, improving the solubility and therapeutic efficacy of sorafenib in hepatocellular carcinoma cells (Figure 9C). This system significantly increased apoptosis and cytotoxicity in cancer cells, demonstrating potential for enhanced cancer treatment. Ray et al. (2019) developed pH-responsive copolymer nanoparticles using mPEG-PLA and iRGD-PEG-PLA for targeted drug delivery. These nanoparticles self-assembled at pH 7.4 and disassembled under acidic conditions, releasing the encapsulated drug or dye. This system enhanced targeted drug release in the tumor microenvironment and endo/lysosomal compartments, improving therapeutic efficacy and drug accumulation (Figure 9D). iRGD has been widely utilized in this field due to its exceptional ability to enhance tumor targeting and penetration. Numerous studies have demonstrated its effectiveness in improving the delivery and therapeutic outcomes of various nanodrug systems (Pei et al., 2022; Lu et al., 2020; Le et al., 2024; Yang et al., 2020).

In conclusion, the recent advancements in RGD-based self-assembled nanodrugs have shown significant promise in enhancing the efficacy of cancer therapies. By exploiting the high affinity of RGD peptides for integrin receptors, these nanodrugs achieve targeted delivery, improved tumor penetration, and reduced systemic toxicity. Multifunctional capabilities, including combined chemotherapy, phototherapy, and imaging, offer comprehensive cancer treatment approaches. Despite the impressive progress, challenges such as optimizing biocompatibility, stability, and large-scale production remain. Continued research and development are essential to address these issues, ensuring that these innovative nanodrugs can be effectively translated into clinical applications, offering improved therapeutic outcomes for cancer patients.

## 4 Challenges and future directions

While RGD-based self-assembled nanodrugs offer significant promise for targeted cancer therapy, several challenges must be addressed to fully realize their potential. One major challenge is immunogenicity and biocompatibility. The body’s immune system may recognize these nanodrugs as foreign, leading to immune responses that can reduce their efficacy and cause adverse effects. Ensuring that these nanodrugs are biocompatible and minimizing their immunogenicity are critical steps to improve patient safety and treatment outcomes. Additionally, the stability of nanodrugs in the bloodstream is a concern, as factors such as protein adsorption, aggregation, and premature drug release can compromise their effectiveness. Developing strategies to enhance their stability, such as surface modifications like PEGylation, can help prolong circulation time and improve targeting efficiency.

Another significant challenge is the heterogeneity of tumors. Variability in integrin expression among different tumor types, stages, and even within the same tumor can affect the targeting efficiency of RGD-based nanodrugs. Addressing this issue may require personalized medicine approaches and combination therapies to enhance treatment efficacy. Moreover, the scale-up and manufacturing of RGD-based self-assembled nanodrugs for clinical use present additional hurdles. The complexity of the self-assembly process and the need for precise control over nanodrug characteristics demand advanced manufacturing techniques to ensure consistency and reproducibility in large-scale production, which is crucial for regulatory approval and clinical application.

Looking forward, several future directions can enhance the development and application of RGD-based self-assembled nanodrugs. Research should focus on improving targeting capabilities by developing multifunctional nanocarriers that combine RGD peptides with other targeting ligands, such as antibodies or aptamers, to enhance specificity and efficacy. Incorporating stimuli-responsive systems that release the drug in response to the tumor microenvironment can further improve targeted delivery. Additionally, improving the biocompatibility and reducing the immunogenicity of these nanodrugs through the use of biocompatible materials and stealth properties like PEGylation will be essential. Personalized medicine approaches that tailor nanodrug formulations to the specific characteristics of a patient’s tumor can enhance treatment efficacy and reduce resistance risks.

Furthermore, combining RGD-based nanodrugs with other therapeutic modalities, such as immunotherapy, radiotherapy, or other chemotherapeutic agents, can provide synergistic effects and improve overall treatment outcomes. Advanced manufacturing techniques, such as microfluidics and nanoprecipitation, will be necessary to ensure the consistent production of high-quality nanodrugs. Finally, extensive preclinical and clinical studies are required to evaluate the safety, efficacy, and pharmacokinetics of these nanodrugs. Rigorous testing in animal models and human clinical trials will provide valuable insights into their therapeutic potential and guide their path to clinical use. By addressing these challenges and pursuing these future directions, the successful clinical translation of RGD-based self-assembled nanodrugs can be achieved, offering improved outcomes for cancer patients.

## 5 Conclusion

RGD-based self-assembled nanodrugs represent a significant advancement in targeted cancer therapy, combining the benefits of nanotechnology with the specificity of RGD peptides. These nanodrugs offer enhanced tumor targeting, increased cellular uptake, and reduced off-target effects, leading to improved therapeutic outcomes. The self-assembly process facilitated by RGD peptides results in stable, efficient nanostructures that protect and deliver chemotherapeutic agents effectively. Despite the promise, challenges such as immunogenicity, stability in the bloodstream, tumor heterogeneity, and manufacturing scalability remain. Addressing these issues through improved biocompatibility, advanced targeting strategies, personalized medicine approaches, and innovative manufacturing techniques is essential for successful clinical translation.

While αvβ3 and αvβ5 integrins have historically been the primary focus of RGD-targeted cancer therapies, recent advances in the understanding of integrin biology suggest that other RGD-binding integrins, such as α5β1 and αvβ6, may offer more selective and efficacious targets for tumor therapy. Future research should continue to explore the diverse roles of these integrins in cancer progression and develop strategies to exploit their unique expression patterns for improved therapeutic outcomes.

Future research should focus on enhancing the multifunctionality of these nanodrugs and conducting rigorous preclinical and clinical evaluations to ensure their safety and efficacy. Incorporating stimuli-responsive systems that release the drug in response to the tumor microenvironment can further improve targeted delivery. Additionally, improving the biocompatibility and reducing the immunogenicity of these nanodrugs through the use of biocompatible materials and stealth properties like PEGylation will be essential. Personalized medicine approaches that tailor nanodrug formulations to the specific characteristics of a patient’s tumor can enhance treatment efficacy and reduce resistance risks.

Combining RGD-based nanodrugs with other therapeutic modalities, such as immunotherapy, radiotherapy, or other chemotherapeutic agents, can provide synergistic effects and improve overall treatment outcomes. Advanced manufacturing techniques, such as microfluidics and nanoprecipitation, will be necessary to ensure the consistent production of high-quality nanodrugs. Finally, extensive preclinical and clinical studies are required to evaluate the safety, efficacy, and pharmacokinetics of these nanodrugs. Rigorous testing in animal models and human clinical trials will provide valuable insights into their therapeutic potential and guide their path to clinical use.

By overcoming these challenges and leveraging the unique advantages of RGD-based self-assembled nanodrugs, we can significantly improve cancer treatment outcomes, offering a more effective and safer therapeutic option for patients. The continued development and refinement of these nanodrugs hold great promise for advancing cancer therapy and improving patient survival rates.
